# Diagnostic Delays and Psychosocial Outcomes of Childhood-Onset Systemic Lupus Erythematosus

**DOI:** 10.7759/cureus.26244

**Published:** 2022-06-23

**Authors:** Afshan Hussain, Marvi V Maheshwari, Nabeeha Khalid, Pragnesh D Patel, Rahmah Alghareeb

**Affiliations:** 1 Research, Dow Medical College and Dr. Ruth K. M. Pfau Civil Hospital Karachi, Karachi, PAK; 2 Research, Our Lady of Fatima University College of Medicine, Valenzuela, PHL; 3 Cardiology, Omar Hospital & Cardiac Centre, Lahore, PAK; 4 Research, St. George’s University School of Medicine, St. George’s, GRD; 5 College of Medicine, University of Baghdad, Baghdad, IRQ

**Keywords:** childhood-onset systemic lupus erythematosus, mental health, presentation delay, morbidity and mortality, diagnostic delay, quality of life, psychosocial outcomes, health-related quality of life, systemic lupus erythematosus

## Abstract

Systemic lupus erythematosus (SLE) is a chronic autoimmune disorder that manifests in affected individuals with a variety of clinical features and involves multiple organs. Despite recent advances over the past decades, higher morbidity and mortality have been reported by studies in patients with childhood-onset systemic lupus erythematosus (cSLE) compared to patients with adult-onset. The interplay of several factors can cause diagnostic delays resulting in worse disease activity, multiple organ damage, increased risk of hospitalization, and management with aggressive treatment. Significant factors include demographic, clinical, and socioeconomic characteristics of patients with cSLE. Moreover, despite recent advances in lupus treatment, prolonged disease duration in these young patients can result in debilitating psychosocial outcomes and can significantly impact their health-related and general quality of life (QOL). Important domains affected include patient self-esteem, education, employment, healthcare utilization, and mental health. In this review, we examined the barriers that lead to a delay in diagnosing lupus in the pediatric population and addressed cSLE morbimortality and its long-term impact on patient health-related and general QOL.

## Introduction and background

Systemic lupus erythematosus (SLE) is a difficult-to-diagnose, chronic autoimmune disorder that involves multiple organs and manifests with a broad spectrum of clinical and immunological features [1,2]. Approximately 20% of patients with SLE present before 18 years of age and get diagnosed with childhood-onset systemic lupus erythematosus (cSLE) [3]. For research studies, ≥4 out of 11 American College of Rheumatology (ACR) criteria for SLE have to be present before or cumulatively at the time of diagnosis for one to be classified as having cSLE [2]. There is limited data on the precise incidence and prevalence of cSLE due to varying cut-offs for diagnosis. Nevertheless, most studies settle on patients receiving a diagnosis before 18 years to be eligible for cSLE [1-3]. A nationwide study on a pediatric SLE population estimated a prevalence of 9.73 per 100,000 children, with 2.22 new cases reported every year [4]. As opposed to adult-onset systemic lupus erythematosus (aSLE), cSLE was five times higher in females than males in contrast to the former, where gender bias is more significant with a 9:1 female-to-male ratio [4,5]. Consistent with aSLE, a higher prevalence of cSLE is observed in ethnic groups other than Caucasians, including Asian Americans, Native Americans, and Hispanics [2,4,6].

Although rare and 10 times less common than aSLE, cSLE has been recognized globally to present with worse disease severity, life-threatening organ involvement, and higher morbimortality [1,2,5,6]. A nested case-control study identified that adolescents are twice more likely to be hospitalized for SLE-associated causes and four times more likely to receive invasive treatment than patients with aSLE [2,6]. cSLE patients are more likely to suffer from renal, hematological, and neurological manifestations [3,6].

Alongside poor mental and physical health, the chronic nature of cSLE is associated with poor health-related quality of life (HRQOL) and high healthcare financial burden for patients and society [7]. Every year in the United States, the estimated cost of cSLE has been calculated to range from $146 million to $650 million [8]. A study conducted in the United States suggested that the cost to care for a child with SLE is three times higher than a patient with aSLE and up to three to four times higher than that of patients diagnosed with juvenile idiopathic arthritis (JIA) [8]. This cost was mainly attributable to the burden of inpatient hospitalization and extensive laboratory investigations to monitor organ damage.

Hence, it is crucial to recognize factors that can cause a delay in diagnosis, contribute to accrual organ damage in cSLE patients, and lead to worse clinical and psychosocial outcomes [4,6,9]. This review serves to assemble factors contributing to a delay in diagnosis in the pediatric population of SLE, including non-specific clinical features, nationality, and socioeconomic background, and explore the morbimortality and the impact of cSLE on quality of life (QOL).

## Review

Morbidity and mortality

Short-term survival rates for SLE have improved over the past decades. Although the disease was once responsible for significant mortality in the 1980s, survival rates have increased to 95% today [9-12]. Studies conducted in the 2000s concluded a five-year survival of 100% and ten-year survival of 90% in cSLE patients [13]. Significant advances in increased disease awareness, earlier recognition of symptoms, and better management of disease and associated comorbidities have improved overall survival in these patients [13-15]. However, despite achieving these critical milestones, the standardized mortality rate remains two to five times higher in adults and approximately 20 times higher in pediatric SLE patients than in the general population [14,16,17].

Patients with cSLE accumulate more organ damage and suffer from worse disease course and outcomes [1-3,6]. It is likely due to increased disease duration and, consequently, more prolonged exposure to harmful immunosuppressive agents [18]. The Systemic Lupus International Collaborative Clinics/American College of Rheumatology Damage Index (SDI) is a reliable tool for measuring cumulative disease damage where damage is referred to irreversible degeneration of an organ or system ongoing for six months [19]. The SDI represents damage in 12 organs or systems with a score ranging from 0 to 47 [19].

In a review, Ravelli et al. determined that almost 60% of cSLE patients suffer from cumulative organ damage [13]. Moreover, these patients accrue disease damage faster than their adult counterparts and are at a higher risk of earlier mortality [6]. Hersh et al. conducted a longitudinal study on 957 patients with aSLE and cSLE and compared mortality rates between the two groups [20]. After a mean follow-up period of 48 months, the authors concluded that cSLE patients had a longer disease duration and were more likely to develop renal manifestations in the ACR criteria for lupus and end-stage renal disease (ESRD). Moreover, deaths in the cSLE cohort occurred at a relatively younger age. After adjusting for multiple covariates, cSLE was an independent predictor of mortality in their study [20].

In 2008, Tucker et al. concluded a two-fold higher mortality rate in cSLE patients [6]. The authors analyzed a multiethnic cohort of 79 lupus patients and compared outcomes between aSLE and cSLE patients after a ≥one-year follow-up [6]. cSLE patients were identified to have a longer disease duration than aSLE patients (6.8 vs. 5.6 years). They were also more likely to develop higher renal and neurological manifestations with a greater extent of renal damage [6]. Renal involvement was defined as World Health Organization (WHO) class II-V in the presence or absence of proteinuria (>0.5 g/24 hours or 3+) attributable to SLE with or without abnormal urinary sediment, proteinuria 2+, elevated serum creatinine/decreased creatinine clearance twice, six months apart [6]. In contrast, a study conducted by Font et al. demonstrated a cohort with more active renal involvement in cSLE patients while more frequent neurological involvement in aSLE patients [21].

With significant improvement in life expectancy, cSLE patients confront an increased risk of morbidity due to disease activity sequel, medication side effects, and comorbid conditions [22,23]. A retrospective study by Brunner et al. on 66 newly diagnosed cSLE patients concluded that disease activity measured by the Systemic Lupus Erythematosus Disease Activity Index (SLEDAI) was the single most important predictor of cumulative damage [22]. Most patients developed damage at the end of the mean follow-up period of 3.3 years, with a mean SDI score of 1.8. The significant domains involved were renal, neuropsychiatric, and musculoskeletal. The use of steroids, acute thrombocytopenia, and the presence of antiphospholipid antibodies were other factors that contributed to disease damage, while the duration of disease management with immunosuppressants protected against damage [22].

Involvement of similar organ systems was demonstrated by Ravelli et al. in a multinational study of 387 pediatric lupus patients recruited from multiple centers across Italy, Greece, the United States, Japan, and Mexico [23]. More than half of the participants had renal and neuropsychiatric involvement, with a mean SDI score of 1.1. Risk factors strongly associated with cumulative damage included lengthened disease duration, neuropsychiatric features at diagnosis, and increased use of intravenous cyclophosphamide pulses [23].

Delays in diagnosis of cSLE

Individuals diagnosed with cSLE are at a higher risk of irreversible damage than their adult counterparts [24,25]. Timely diagnosis, earlier referral to sub-specialists, and aggressive management improve long-term outcomes, prevent organ failure, and increase survival in these patients [26-29]. A study conducted in the United States indicated that cSLE patients were less likely to present without delay if they lived in a poor density area for pediatric rheumatologists [28]. Approximately a quarter of the US pediatric population has to travel more than 80 miles to reach the closest pediatric rheumatologist [30]. Over one-third of pediatricians in medically underserved areas reported referring cSLE patients to adult rheumatologists due to specialist shortages demanding long hours of travel or lengthy waitlists [31]. Access to sub-specialists is delayed by geographical distance, limited provider volume, and high patient burden [28]. However, studies from relatively small countries showed no significant association between geographical distance to the nearest sub-specialist facility and delayed diagnosis [29,32].

Late diagnosis could pose a more significant severity threat to cSLE patients in developing nations due to scarcity of medical resources, shortage of sub-specialists such as pediatric rheumatologists, and lengthy waitlists. There is a projected requirement of at least 40 pediatric rheumatologists with currently registered specialists to provide adequate care for over 390 million children in South Africa [33].

Lewandowski et al. conducted a study on the South African population and identified barriers to timely diagnosis through semi-structured interviews of 22 primary caregivers of patients diagnosed with cSLE [34]. These patients met four out of eleven ACR criteria and were diagnosed before 19 years of age. The mean time to diagnosis from symptom onset was 23.5 weeks. The interviews were recorded and analyzed for common themes in delayed diagnosis classified into either caretaker-related or health system-related barriers [34]. Factors impeding timely diagnosis of cSLE under caretaker barriers included lack of symptom recognition, confusing non-specific symptoms with benign etiologies, considering medical evaluation only with worsening health status, financial difficulties of hospital bills, transport, and missed days of work, and SLE being misunderstood as a contagious disease causing fear of social stigma [34]. Health system-related hurdles included lack of training among first medical contact providers, families being sent home without proper communication or appropriate clinical workup, complex medical systems requiring multiple visits before obtaining a formal referral to a sub-specialist, and misdiagnosis of SLE [34]. Numerous referrals before reaching a sub-specialist is an essential factor leading to delayed diagnosis of cSLE [25,28,29,32]. Lewandowski et al. indicated that 63% of patients at the time of diagnosis had severe symptoms such as pericarditis, acute renal failure, and stroke and required intervention [34].

Neglected disease owing to delay in initiating treatment manifests with worsened disease severity and outcomes. An Indian study showed high mortality of 32% in patients with cSLE followed over 10 years [35]. The majority of patients in the study had died at or after the first admission to the hospital due to uncontrolled disease activity attributable to delayed diagnosis and late referral to a specialist facility more equipped to initiate prompt therapy [35].

A meta-analysis by Tektonidou et al. examined trends in survival from 125 adults and 51 pediatric studies conducted on lupus patients, each from low-middle and high-income countries between 1950 and 2016 [25]. The study reported a five-year survival of over 95% among both aSLE and cSLE participants belonging to high-income countries. However, a lower five-year and ten-year survival was concluded among the children (85% and 79%, respectively) compared to adults (92% and 85%, respectively) belonging to low-middle income countries. The insufficiency of sub-specialists and limited healthcare resources contributed to this imbalance [25].

In further support, a study by Jongvilaikasem et al. retrospectively analyzed survival trends in cSLE patients in Thailand over three decades [26]. The authors determined that pediatric cases receiving a lupus diagnosis after 2006 had 2.5 times better outcomes than those with an incidence in earlier decades. This improved prognosis was ascribed to amiable access to healthcare in Thailand, improved quality and availability of medical resources, increased cSLE awareness, and earlier recognition by primary care physicians with prompt referrals to sub-specialists [26].

Furthermore, to determine clinical and demographic differences leading to a delayed presentation among cSLE patients, Rubenstein et al. conducted a study on 598 lupus patients from Childhood Arthritis and Rheumatology Research Alliance (CARRA) Legacy Registry [28]. Patients were stratified according to the time from symptom onset to the first presentation to a pediatric rheumatologist [28]. Factors related to early presentation (<one month), moderate delay (one to three months), and severe delay (>one year) were determined by multiple logistic regression. The median time from symptom onset to the first presentation to a pediatric rheumatologist was 1.4 months for the overall participants. The study revealed that patients with earlier presentation (44%) were associated with residence in a state with a high density of pediatric rheumatologists, positive family history of lupus, and an absence of discoid rash. In contrast, patients with severe delay (9%) were associated with low household income, younger age, and positive family history. The study concluded that increased delay was inversely related to the age of onset and fulfillment of ACR criteria for lupus while directly related to low socioeconomic family background and annual family earnings of <$25,000 [28]. A positive family history of lupus was associated with the earliest and latest presenters in the study [28]. The authors suggested that familiarity with diagnosis and a pre-existing alliance with rheumatologists may have led to a timely presentation. In contrast, the burden of caring for an additional affected family member with lupus and preferred consultation with an adult rheumatologist may have served as reasons for the cases latest in presentation [28].

In contrast, a study conducted in the United Kingdom revealed that variables such as age at symptom onset, financial status, or family history of autoimmune disease did not significantly predict delays in the care of cSLE patients [32]. Smith et al. analyzed data from 257 patients recruited in UK JSLE (juvenile-onset SLE) Cohort Study from multiple specialist centers across the United Kingdom between 2006 and 2011 [32]. The study concluded a median time delay from symptom onset to cSLE diagnosis of 0.4 years. A non-White ethnicity (Asian, African, or Caribbean), referral to a pediatric rheumatologist by a pediatrician, and nephritis were identified as independent predictors of shorter time to cSLE diagnosis. At the same time, age at symptom onset, sex, ACR score, geographical distance to the nearest sub-specialist facility, financial status, or family history of autoimmune diseases was not significantly associated with delay in care [32].

Reversible damage due to the underlying inflammatory process of SLE is referred to as disease activity [36]. Worsening disease activity that can be measured as new or deteriorating clinical signs and symptoms in at least one organ system is termed a flare [36]. Severe flares require more aggressive treatment [2,36]. In comparison, disease damage in SLE is irreversible degenerative changes on tissue or organ level due to underlying disease activity or response to lupus treatment [36]. The SLEDAI and European Consensus Lupus Activity Measurement (ECLAM) are frequently used scales to measure cumulative disease activity in cSLE [36,37]. Although both scales can similarly predict disease damage and the need for steroids, SLEDAI is preferred for its ease and popularity for use in patients with aSLE [36,37]. Higher scores on these scales correlate with increased tissue injury and mortality [22].

A retrospective cohort study in Brazil divided 1,555 cSLE patients recruited from 27 rheumatology services into three groups based on time to diagnosis: group A: short time interval (<one month), group B: intermediate time interval (one to three months), and group C: long time interval (>three months) [27]. Compared to groups B and C, participants in group A (4%) were associated with higher frequencies of worse disease activity scores, severe initial multiorgan manifestations such as palate ulcers, serositis, nephritis, hematological and neuropsychiatric involvement, thrombocytopenia, leukopenia/lymphopenia, and higher titers of anti-dsDNA antibodies. In contrast, group C participants (62.5%) forming the largest group in the study abundantly presented with milder initial symptoms such as synovitis and had lower frequencies of more severe signs such as serositis, and proteinuria >500 mg/24 hours, and low complement levels. This large-scale Brazilian study concluded that diagnosis is delayed in most cases of cSLE due to mild disease onset, while severe symptoms aid earlier recognition of SLE in a minority of patients and subsequent timely referral to pediatric rheumatologists [27].

Additionally, Cervera et al. conducted a multicenter study across different countries of Europe using a Euro-lupus cohort composed of 1,000 SLE patients prospectively followed for 10 years since 1991 [24]. In total, 76 patients in the cohort (8%) were diagnosed before 14 years of age and were termed cSLE patients. A mean delay from initial symptom manifestation to SLE diagnosis was two years in adults compared to five years in cSLE patients. Most patients in the cohort presented with non-specific symptoms such as arthritis and fever, while classic SLE malar rash was reported in fewer than 50% of patients. The study revealed that practitioners were unwilling to diagnose SLE in children due to non-specific signs and symptoms and were less likely to pick up on milder disease features in the pediatric population [24].

Lukic et al. also analyzed the incidence, clinical, and laboratory features in 81 children with cSLE in Croatia between 1991 and 2010 and retrospectively identified factors causing a delay in diagnosis [29]. The median time delay from symptom onset to diagnosis among study participants was two months. The results demonstrated an inverse relationship between time to diagnosis and increased disease severity determined on the ECLAM scale. At the same time, no significant association was found with age, gender, distance from the closest sub-specialist facility, or clinical, demographic, and laboratory features of study subjects. This large-scale study concluded that earlier recognition of children with fewer and less severe symptoms of SLE would improve prognosis and avoid serious disease consequences in these patients [29].

Similarly, in a study with a prospective multinational design, Gomez et al. evaluated and compared disease characteristics in patients with cSLE (n = 230) and aSLE (n = 884) recruited from the Grupo Latino Americano de Estudio del Lupus (GLADEL) cohort from nine countries in Latin America [38]. Although not significant, the time for diagnosis among the study participants was less in the pediatric group than in the adult group, with a median delay of 129.5 days versus 186.5 days. The study revealed that severe disease features in cSLE patients decreased time to diagnosis due to the rapid fulfillment of ACR criteria, higher disease severity scores at diagnosis, and presentation with more frequent manifestations of fever, mouth ulcers, malar rash, neurological, and hematological abnormalities. Adult participants presented more frequently with myalgias, cranial nerve involvement, and Sjogren’s disease. These findings led the authors to conclude that patients with cSLE presented with higher disease activity scores and significant multiorgan involvement than patients without aSLE [38]. Differences in clinical manifestations were also identified among ethnic groups in the study; a higher prevalence of fever, thrombocytopenia, and hemolytic anemia was found in Afro-Latin-American (ALA) children, while neurological features (cerebrovascular disease, cranial nerve involvement) were more prevalent in Mestizo children [38]. cSLE patients in the UK cohort study also recognized earlier symptom recognition in Asian, African, and Caribbean patients [32].

Alongside mild disease features, atypical symptoms in cSLE cases can significantly delay diagnosis [39]. In a multicenter French study, 32% of cSLE patients presented with atypical symptoms, predominantly abdominal, leading to surgical intervention in three cases for presumed appendicitis before a cSLE diagnosis was established [40]. Likewise, a cross-sectional study in Brazil that recruited 852 subjects with cSLE identified that 3.7% of these patients presented with chronic arthritis (CA), mainly in the hands and knees [41]. In more than half of these cases, the initial diagnosis was JIA, delaying lupus diagnosis by up to a month from initial joint symptoms. The study characterized CA as a rare and early manifestation of cSLE, frequently mimicking JIA at disease onset and associated with significant musculoskeletal accrual damage [41]. Late recognition of lupus as an underlying etiology of arthritis manifestations in these patients may cause restrictive movement behaviors of the affected joints and result in shortened tendons and contractures [29].

Table 1 gives details of some clinical studies that identify factors contributing to a delay in cSLE diagnosis [24,25,27-29,32,34,38,41], all of which are mentioned in this review.

**Table 1:** Summary of included studies identifying factors contributing to the delay in the diagnosis of cSLE. cSLE: childhood-onset systemic lupus erythematosus; SLE: systemic lupus erythematosus

References	Country/Region	Design	Study sample	Time from symptom onset to diagnosis of cSLE	Conclusion
Novak et al. (2018) [27]	Brazil	Retrospective cohort study	1,555 patients with cSLE	Three delay groups: A: short time interval (three months)	Mild disease symptoms increased delay in diagnosis of cSLE
					Severe disease manifestations with multisystemic organ involvement decreased the time to diagnosis for cSLE
Rubinstein et al. (2018) [28]	United States	Retrospective cohort study	598 patients with cSLE	1.4 months	A family history of SLE and living in a high-density area with pediatric rheumatologists resulted in the early presentation of cSLE patients
					A family history of SLE, young age of onset, and low household income delayed presentation of cSLE patients
Tektonidou et al. (2017) [25]	-	A systematic review and Bayesian meta-analysis	125 adult, and 51 pediatric studies conducted on SLE patients	-	Barriers to healthcare access and limited availability of experienced clinicians increased the time to diagnosis for cSLE patients from low-income countries
Lewandowski et al. (2017) [34]	South Africa	Qualitative semi-structured interviews	22 caregivers of patients with cSLE	23.5 weeks	Lack of awareness, social stigma regarding SLE, financial difficulties, lack of trained staff, misdiagnosis, and complex medical system increased the time to diagnosis for cSLE
Sakamoto et al. (2016) [41]	Brazil	Cross-sectional study	852 patients with cSLE	1 month	Chronic arthritis as an initial manifestation increased the time to diagnosis in cSLE
Smith et al. (2014) [32]	United Kingdom	Cohort study	257 patients with cSLE	0.4 years	Ethnicity, referral by a pediatrician, and presence of lupus nephritis decreased the time to diagnosis in cSLE
Lukic et al. (2013) [29]	Croatia	Retrospective study	81 patients with cSLE	2 months	Increased disease activity decreased the time to diagnosis in cSLE
Cervera et al. (2009) [24]	Europe	Prospective cohort study	76 patients with cSLE out of 1000 SLE patients	5 years	Non-specific symptoms and mild disease activity increased the time to diagnosis in cSLE
Gomez et al. (2008) [38]	Latin America	Prospective cohort	230 children with SLE	129.5 days	Higher disease severity decreased the time to diagnosis in cSLE patients

Health-related quality of life

Despite similar improvement in survival patterns for cSLE and aSLE, not much is known about the long-term outcomes of cSLE patients once they reach adulthood [13,42-44]. Adaptations made for a long-standing disease may impair the physical and psychological aspects of a patient’s life. Increased disease activity with more significant renal damage necessitates more aggressive treatment and management in cSLE patients, twice increasing the risk of hospitalization and intravenous therapy that may influence HRQOL in lupus-afflicted adolescents [6].

Ruperto et al. determined that the involvement of central nervous, renal, and musculoskeletal systems had the most impact on HRQOL in cSLE patients, with the occurrence of seizures, active nephritis, and bodily pain reported to cause significant interference in family life [45]. Measurement of HRQOL is imperative to account for the physical, emotional, and social support required by patients alongside treatment for SLE [13,45]. The Federal Food and Drug Administration (FDA) has suggested taking HRQOL into account with traditional laboratory and clinical outcomes to assess the impact of treatment [46]. Therefore, providers should use valuable health resources to ensure a management with well-rounded benefits to the patients, including an increase in HRQOL alongside keeping disease activity at bay [46].

Studies have evaluated the HRQOL of patients with cSLE [45,47,48]. Ruperto et al. conducted a cross-sectional, multicenter study on 297 patients with cSLE [45]. HRQOL of these subjects was compared to children with other chronic rheumatic diseases and healthy controls using the Child Health Questionnaire (CHQ) [45]. CHQ is an instrument used to assess physical, emotional, and social aspects of health in children over five years of age [45]. Overall, cSLE patients demonstrated lower scores in physical and psychosocial domains of HRQOL, with physical health being more prominently affected [45]. The most affected subscales on the CHQ were identified to be global health, general health perception, and parent impact-emotional. Moreover, disease activity measured by SLEDAI correlated with both physical and psychosocial summary scores on CHQ, while accumulated damage measured by SDI was significantly associated with the physical summary score component [45].

Similarly, in a multicenter cohort study, Brunner et al. reported that organ-specific disease activity, measured by the British Isles Lupus Activity Group Index (BILAG), was associated with lower HRQOL in children with SLE [47]. Higher BILAG scores in specific domains, especially general (mostly fatigue), musculoskeletal (mostly arthralgias and arthritis not limiting function), neurological (mostly headaches and migraines), and vascular (mostly Raynaud’s phenomenon), were associated with a significantly lower HRQOL. In contrast, relation with other domains of BILAG such as mucocutaneous, renal, and cardiovascular was not statistically significant [47].

In contrast, in a cross-sectional study, Jones et al. concluded that drug therapy targeting suppression of disease activity and damage alone was insufficient to improve HRQOL in cSLE patients [48]. The authors suggested a focus on combined clinical and psychological aspects to result in better outcomes in these patients [48]. Decreased HRQOL in study subjects was also associated with pain, fatigue, depression, and anxiety, with a prevalence of 40%, 65%, 30%, and 37%, respectively [48]. Fatigue has been reported as a significant symptom by young patients with SLE [49].

The neuropsychiatric manifestations in SLE commonly include headache, cerebrovascular disease, chorea, new-onset seizures, and mood disorders [40]. These manifestations and the general disease burden adversely affect mental health and often go overlooked [2]. Mental health problems such as anxiety, depression, and suicide ideation are frequent in the cSLE population (Table 2) [48,50,51].

**Table 2:** Mental health characteristics: depression, suicidal ideation, and anxiety prevalence in cSLE patients. ^1 ^Symptoms screened in [50] and [51] by Patient Health Questionnaire-9 (PHQ-9); ^2^ Symptoms screened in [48] by Children’s Depression Inventory Version I (CDI-I) scale; ^3^ Symptoms screened by Screen for Childhood Anxiety Related Disorders (SCARED) questionnaire. cSLE: childhood-onset systemic lupus erythematosus

	Knight et al. (2014) [50]	Jones et al. (2017) [48]	Davis et al. (2019) [51]
Number of study subjects	50	60	51
Depression,^1,2^ N (%)	10 (20)	18 (30)	30 (58.8)
Suicidal ideation, N (%)	7 (14)	-	7 (13.7)
Anxiety,^3^ N (%)	11 (22)	22 (37)	-

In a cross-sectional study, Knight et al. reported that depression and anxiety symptoms were prevalent in one-third of their subjects, with suicide ideation present in 14% [50]. Of importance, only 24% of these patients reported seeking mental health care for their symptoms, lower than the 31.4% of study subjects reported by Davis et al. [50,51]. According to a qualitative study conducted in the United States, barriers to receiving mental health included stigmatization, fear, the emotional burden for parents, uncertainty about getting help, minimization by doctors, and limited access to mental healthcare [52]. In contrast, factors that encouraged seeking mental help included strong relationships with the clinicians, initiative of screening by providers, increased patient and family awareness, and normalization of mental health issues [52]. Obstacles in seeking mental healthcare should be addressed in this population to improve QOL and treatment outcomes [50].

Due to the annual recommendations for depression and anxiety screening, patients with cSLE are most likely to be identified by their pediatricians in outpatient clinics [53]. Young patients with SLE and their families prefer pediatric rheumatologists as a source for mental health screening, guidance, and referral [52]. However, while 77% of pediatric rheumatologists in a study acknowledged the importance of routine depression screening in cSLE patients, only 2% reported screening completion using standardized tools and protocols, citing limited staff resources and time as the most frequent barriers to routine screening [51]. Moreover, a study by Chang et al. identified that medication adherence in SLE youth increased in patients who received treatment for psychiatric disorders [54]. Likewise, in a cross-sectional study, Davis et al. concluded that the severity of depression symptoms was directly related to medication non-adherence among cSLE patients [51]. Therefore, it is imperative to recognize psychiatric disorders in this population to improve adherence and increase positive outcomes.

Long-term outcomes

While equally likely to maintain romantic relationships, marry, and live independently, young adults suffering from childhood-onset heart disease, diabetes, malignancy, and epilepsy face poor educational and vocational outcomes and are more reliant on public assistance compared to the general population [55,56]. Challenges of adolescence compounded by chronic illness may bring about many more hurdles for young SLE patients than their healthy peers [57]. Disease features such as arthritis and cognitive dysfunction may cause pain and difficulty in school-related activities. Changes in physical appearance due to disease features (discoid rash, malar rash, alopecia) and treatment side effects with high-dose steroids (stria, weight gain, moon facies, acne) and others (infertility) prevent patients from adhering to their treatment regimens [45,57]. They may also develop poor self-esteem and perceived body image [45,57].

Domains of QOL impacted by lupus were identified in a qualitative study by Moorthy et al. [58]. Single open-ended questions were asked from children with SLE and their parents. Responses by children were mostly along with themes of coping and maintaining control of their lives despite a chronic illness. On the other hand, responses from parents were based on dealing with their child’s disease and displaying appreciation for their child’s coping process [58]. Parents of the study subjects in Ruperto et al. were also concerned about their child’s overall health, susceptibility to illness, and emotional, psychological, and social well-being [45].

Adults suffering from a childhood-onset chronic illness may experience limitations in reaching important social, educational, and vocational milestones compared to healthier individuals. Lower education status and employment rates put them at risk of poor health insurance coverage and subsequent delayed healthcare access [59,60]. After a 13.6-year follow-up of 64 cSLE patients, Chalom et al. determined their vocational, socioeconomic status, and QOL [61]. About one-third of patients in the study reported that their chronic illness interfered with their education. A quarter of these participants had full-time jobs, 22% had part-time employment, and, overall, the study subjects’ income was low [61]. These figures were worse than those reported by JIA patients [62,63].

Interestingly, in a nine-year follow-up study, Lawson et al. determined that in comparison to aSLE patients, cSLE patients were equally likely to complete at least a bachelor's degree but significantly less likely to enter work and gain employment regardless of demographics and disease features [64]. Moreover, over the study period, a higher proportion of cSLE participants developed renal disease and became candidates for dialysis, further decreasing the likelihood of employment [64]. Legislative programs such as Section 504 of the Individuals with Disabilities Act and the Individuals with Disabilities Education Act fulfill special healthcare needs and ensure successful completion of education milestones in populations requiring additional social support [64]. However, these individuals cannot transition to a dynamic work environment because they lack training and support. cSLE subjects are more likely never to enter work than subjects with disease onset in adulthood [64]. Consequently, lower employment rates impair chances for employer-based health insurance coverage, which is currently the most prevalent source of insurance for non-disabled adults [65]. Hence, programs offering vocational training to students with chronic illness may better prepare them for challenging work environments and provide training to maximize their potential, attain suitable employment, and afford insurance [66].

Most young patients’ view of chronic illness interferes with reaching their maximum physical and social capacities. It hinders them from creating personal and career goals more than their healthy peers [58,67,68]. Despite having no behavior problems and an intact ability to socialize with others, cSLE patients are usually targets of uncertain identities, poor self-esteem, and stigmatization by peers and teachers [45]. Moreover, they frequently report worrying about their future [45]. Studies showed that these patients scored lower on QOL surveys, particularly in the domains of physical health, school performance, worry, and self-esteem than healthy subjects and children with arthritis [58,68].

In a retrospective cohort study, SLE onset at younger age, less social support, and lower self-efficacy for disease management (the belief that one can control their disease) were linked with higher disease activity [69]. Lower self-efficacy was also significantly associated with poor physical function and mental health [69]. Poor social support and disease awareness aggravates feelings of insecurity and lowers confidence in cSLE individuals regarding their ability to self-manage their chronic illness [67,70]. Psychosocial and educational interventions such as public and private forums may promote disease awareness, self-management, and self-efficacy. They may also improve QOL and disease outcomes in these young patients with cSLE [67,71].

Limitations

Majority of the studies included in this review focus on essential research conducted on cSLE patients. Except for comparison purposes, extensive data about SLE in adults has not been added, and only studies with a free version online were included. Furthermore, HRQOL and QOL are subjective concepts hard to measure in continuously developing children. In most qualitative studies included in this review, HRQOL and QOL were measured by scores adapted from adult scales and not specifically designed for children with lupus, which may invalidate some results depending on the participant’s cognitive and developmental level.

## Conclusions

Although rare, cSLE presents with worse clinical outcomes and impact on QOL in affected individuals due to its chronicity, complexity, and many presentations. This review has addressed the interplay of patients and healthcare-related factors that serve as barriers leading to a delayed diagnosis and care in cSLE. Evidence suggests that a prompt diagnosis is necessary to prevent disease-related mortality and morbidity in these patients. We believe this can be accomplished by developing screening guidelines that effectively rule out or rule in cSLE, especially when children present with mild or atypical disease features, and implementing policies that improve healthcare delivery in medically underserved areas.

We have also emphasized the psychosocial challenges these children and adolescents face as they grow older and interact with society. The transition of life challenges and battling a chronic illness can impact significant aspects and QOL in young patients with lupus. This article highlights the critical need for developing interventions that improve social support such as education, employment, and health insurance in this population.

We believe physicians can play a vital role in the holistic management of cSLE patients by tackling disease activity and creating a trustworthy alliance with patients and families. Providers should also empower patients with knowledge about their condition and the ability to self-manage. Furthermore, efforts should be made to increase disease awareness among the public to minimize stigma, promote tolerance, and earlier recognition of symptoms in both family and clinical settings. Finally, we encourage more research to identify the impact of cSLE on affected individuals and society and explore solutions that improve long-term outcomes in this population.
